# New is not always better: Virtual reality does not necessarily enhance mnemonic processing

**DOI:** 10.3389/fpsyg.2023.1089725

**Published:** 2023-02-15

**Authors:** Marike Johnsdorf, Kim Anh Pham, Tino Schmidt, Van Ly Truong, Andre Wohnig, Joanna Kisker, Thomas Gruber, Benjamin Schöne

**Affiliations:** Experimental Psychology I, Institute of Psychology, Osnabrück University, Osnabrück, Germany

**Keywords:** memory, virtual reality, learning, technology, education

## Abstract

Integrating new technologies such as Virtual Reality (VR) can contribute to increasing efficiency in several areas relevant to society. VR can be applied in various contexts and has the potential to improve mnemonic processes and memory performance. However, the specific conditions under which VR is more beneficial than conventional learning methods remain unclear. To further investigate the value of VR for mnemonic processing, participants performed a memory task under three different conditions. For that task, they were presented with rules regarding the spatial arrangement of building blocks with a written text or a video in 2D on a screen or in 3D/360° with a head-mounted display. Following the learning session, memory performance was measured by a recognition test involving a multiple-choice questionnaire, in which participants had to mark the correct arrangement of building blocks, and a construction test, in which they had to arrange five different building blocks according to the rules learned. Additionally, participants had to arrange 38 building blocks according to the rules in a free recall test the following day. Surprisingly, results revealed no superiority effect for learning in VR. Instead, learning the rules with the text yielded the best memory performance results, indicating that prior experience with conventional learning methods facilitates declarative knowledge acquisition. Considering previous findings regarding cognitive processing in VR, our results suggest that in passive learning, processing the more salient and personally relevant virtual stimuli in the surrounding VR environment requires more attentional resources. Therefore, VR impairs focusing on the relevant declarative information and impedes the transfer of the learned knowledge to different contexts. When considering to implement VR, the value to the particular domain and specific learning task should be taken into consideration: For learning basic declarative information without actively involving the students, conventional learning methods seem sufficient and more efficient for mnemonic processing compared to new technologies.

## 1. Introduction

Technology plays a fundamental role for economic growth by increasing efficiency in various areas relevant to society, such as education (Raja and Nagasubramani, 2018). Therefore, using the potential of rapidly emerging new technologies for improving learning in education has been a recurring subject of research in recent decades (e.g., Harel and Papert, 1990; Schacter, 1999; Culp et al., 2005; Wang et al., 2010; Woolf, 2010; Flavin, 2016). Integrating technology in education can enhance learning as well as teaching styles (for review see Saidin et al., 2015). In particular, it can foster students’ engagement (Bond et al., 2020) and has the capability to increase learning outcomes when combined with effective learning principles (for review see Yeung et al., 2021). One technology that is becoming increasingly relevant in educational contexts is Virtual Reality (VR), which provides highly immersive, i.e., surrounding and interactive (Sanchez-Vives and Slater, 2005), virtual learning environments (Radianti et al., 2020). VR can be applied in multiple learning contexts, ranging from skill acquisition to visualization to cognitive learning in different domains (for review see Hamilton et al., 2021). VR provides various learning contents that normally would not be accessible (Elmqaddem, 2019), and, in comparison to conventional learning methods, VR can improve learning by increasing the learner’s engagement, interest, and motivation (Checa and Bustillo, 2020). From an educational perspective, several theories regarding aspects enhancing effective learning in virtual environments have been developed. For example, one current model takes the influence of affective and cognitive factors and the interaction between media and method on learning outcomes into account (Makransky and Petersen, 2021). These models provide presumptions about the effectiveness of different virtual learning environments with respect to the learning method. However, the basic mnemonic mechanisms underlying the learning processes in educational contexts require further research. Several studies on these mnemonic mechanisms have observed an advantage of VR for memory processes as well. Specifically, several studies found a beneficial effect of VR on episodic memory performance compared to other technologies (for review see Smith, 2019). For example, immersive virtual in comparison to computer-based two-dimensional screen experiences resulted in superior memory performance regarding the recollection of scenes from the respective experience (Schöne et al., 2019). However, findings regarding memory superiority for VR in comparison to 2D are inconsistent, as it could only be observed during free recall but not during cued recall tasks (Schöne et al., 2021a), or as no differences in memory performance between VR and 2D video conditions could be observed at all (Kisker et al., 2021c). Despite the inconsistencies regarding memory performance, there is clear evidence for distinct memory processes in VR compared to conventional computer-based setups. Memories obtained from a VR experience are more vivid and effortlessly retrieved than those from 2D experiences (Kisker et al., 2021c) and become part of a complex associative network resembling autobiographical memory (Schöne et al., 2019). Whether these qualitatively distinct memory processes in VR (Kisker et al., 2021c) promote better memory performance than memory processes during the use of computer-based applications depends on several factors, such as the level of immersion, the visual fidelity, or the potential interactivity with the environment (for review see Smith, 2019). However, the specific conditions under which VR may have a valuable advantage for general mnemonic processing underlying learning over conventional learning methods have yet to be defined.

While many VR applications in education have previously been evaluated regarding usability rather than learning outcomes (Radianti et al., 2020), this study focused on comparing memory performance as a learning outcome across different learning methods. To this end, participants were presented with identical information using either a conventional written text, a 2D video on a screen, or a 3D/360° video in VR. Within these conditions, participants were instructed to memorize presented rules regarding the correct spatial arrangement of building blocks, immediately followed by recognition tests and a free recall test the following day. Except for the two- or three-dimensionality and the presentation *via* screen or head-mounted display, the videos were identical and their content corresponded to the text. Whereas many studies investigating memory performance involved active learning conditions within complex environments (e.g., Krokos et al., 2019; Kisker et al., 2021a), we used a learning context that focused on the cognitive acquisition and memorizing of information without additional tasks and impressions. This study design allows for a direct comparison of the memory performance measured by the recognition and free recall tests and provides information about the usefulness of each method for effective retrieval while reducing the number of influencing variables. Since the transferability of learning content to real-life tasks is crucial for effective real-life application (Hung, 2013), the free recall test was conducted under real-life conditions, i.e., the learned information had to be applied in constructing real-life building blocks, independently from the modality used during the learning session. Therefore, differences in memory performance can be directly associated with the respective learning condition.

Considering the potential positive influence of new technologies on mnemonic processes, we expected to observe better memory performance for technology-based learning as opposed to learning with a written text. Additionally, due to the distinct mnemonic processes in virtual as opposed to computer-based environments (Schöne et al., 2019; Kisker et al., 2021b,c), we also expected to observe different levels of memory performance during the memory tasks between these two conditions. Since the task involves the spatial arrangement of three-dimensional building blocks, the three-dimensionality of the VR environment should be beneficial for learning the rules, improve the transferability and therefore increase task performance. Overall, different outcomes in memory performance between the conditions could yield further insight into the factors influencing learning and memory performance and the contexts in which the respective methods can most successfully be applied.

## 2. Methods

### 2.1. Participants

Eighty-eight participants from various fields of study were recruited from Osnabrück University and received partial course credit or 5€ for their participation. They were screened for psychological and neurological disorders, and normal or corrected to normal vision was required, while glasses were not allowed. The study was conducted in accordance with the Helsinki Declaration and was approved by the local ethics committee of Osnabrück University. Participants gave informed written consent.

The participants were randomly assigned to the three conditions (VR, video, text). Due to missing data, six participants had to be excluded. Therefore, the data of 82 participants were included in the analysis (18 male, 64 female; VR: *N* = 26, *M*_age_ = 21.54, *SD* = 2.16; Video: *N* = 28, *M*_age_ = 22.00, *SD* = 3.54; Text: *N* = 28, *M*_age_ = 21.18, *SD* = 2.76).

### 2.2. Stimulus material and procedure

**Figure 1:**
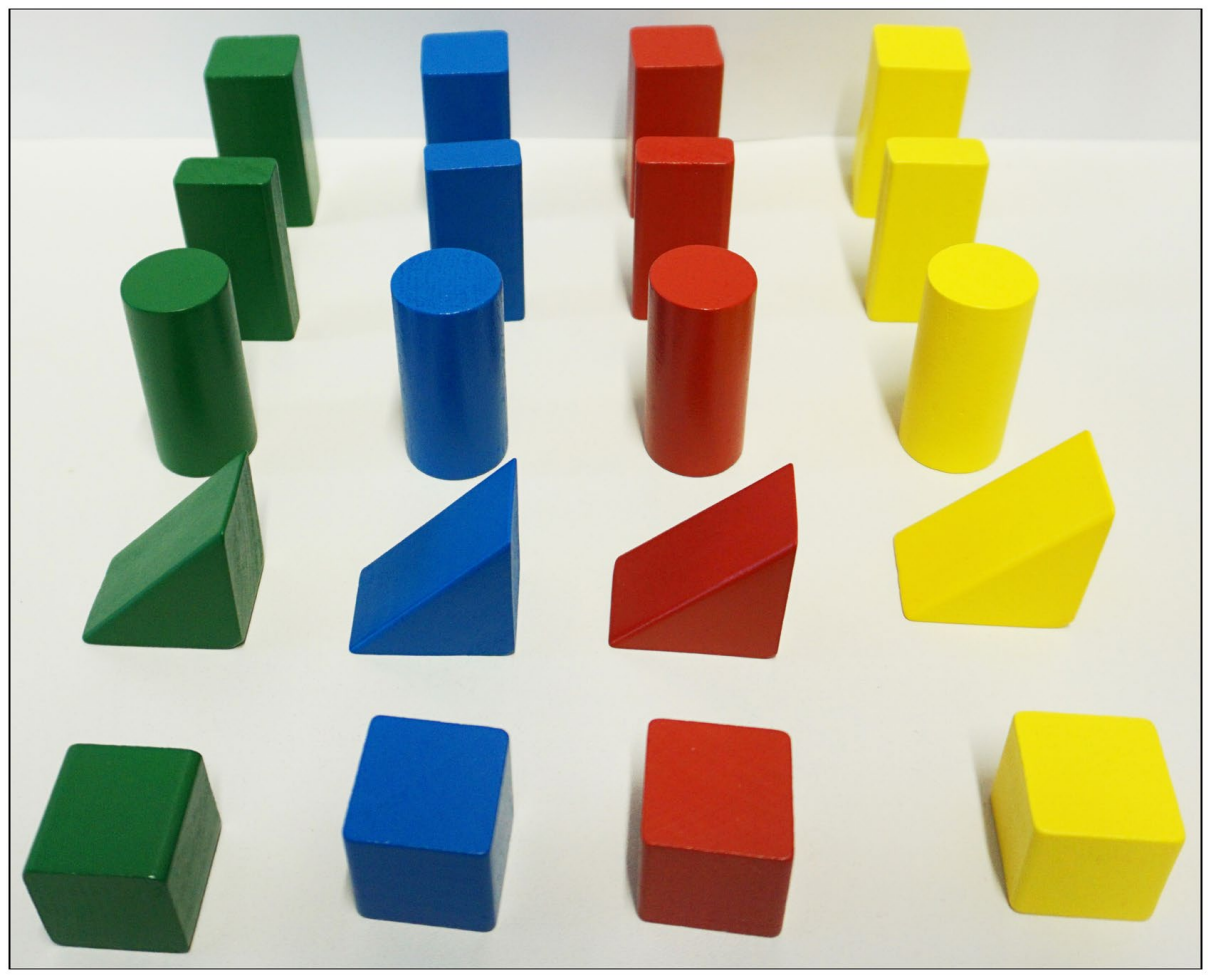
Stimulus material: Building blocks in 5 different shapes and 4 different colors.

The study was conducted on two consecutive days. On the first day, participants learned rules for the spatial arrangement of building blocks *via* text, a 2D video on a screen, or a 3D/360° video in VR. The building blocks differed in shape (cuboids, flat cuboids, cylinders, tetrahedrons, cubes) and color (yellow, blue, green, red; see Figure 1). The rules consisted of two independent sets of 5 rules each (set A, set B, see Supplementary Table S1), which differed in complexity. The rules referred to the positioning of the building blocks in terms of shape and color (e.g., rule 1: “Cuboids/Flat cuboids have to stand on their smallest side.“, for further details see Supplementary Table S1).

**Figure 2:**
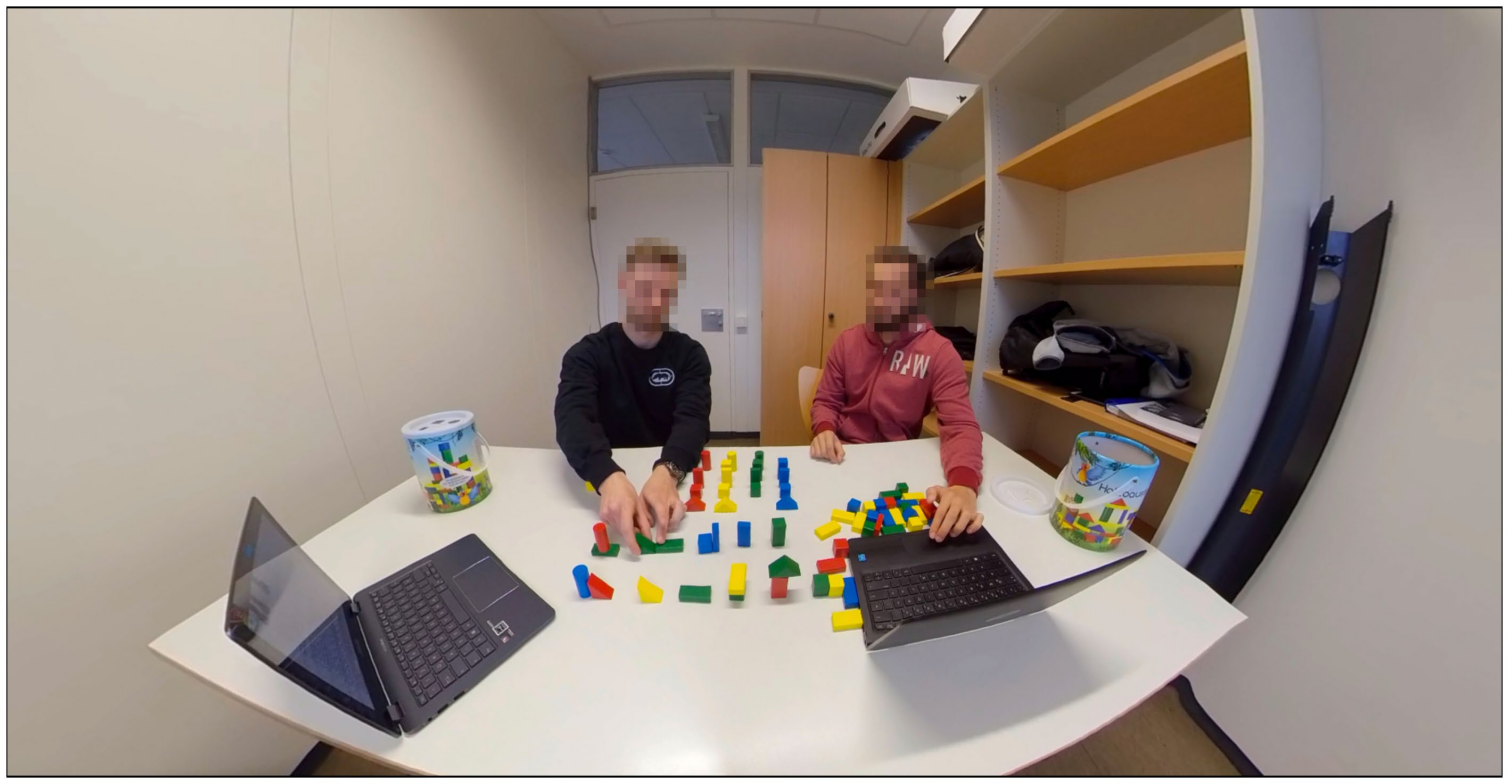
Stimulus material: The recorded video material was used for both the 2D and the 3D/360° video.

In the learning session, participants in all conditions received the same information about the rules. The video material used in the VR and 2D conditions was recorded with the Insta360Pro 3D/360° camera with a resolution of 4 k (3840 × 2160 pixels). The video was stitched with the Insta360Stitcher,^1^ resulting in the same video in 3D/360° for the VR condition and in 2D for the video condition (see Figure 2). In the VR condition, the video was presented with the HTC VIVE Cosmos (with a resolution of 1440 × 1700 pixels per eye), and the 2D video was presented on a conventional screen (17″, resolution of 1920 × 1080 pixels). In the videos, the rules for the arrangement of the building blocks were demonstrated by two instructors. They briefly presented the different shapes and colors of the building blocks and explained each rule while arranging the building blocks according to the respective rule. All rules were presented a second time, resulting in a video length of 5 mins. The same wording as in the video was used for the instructions in the text condition, which were additionally illustrated with pictures. According to the video length, participants in the text condition had 5 mins to learn the rules. Apart from the respective stimulus material, no additional learning material was provided to any of the participants and no instructions for applying specific learning methods were given.

**Figure 3:**
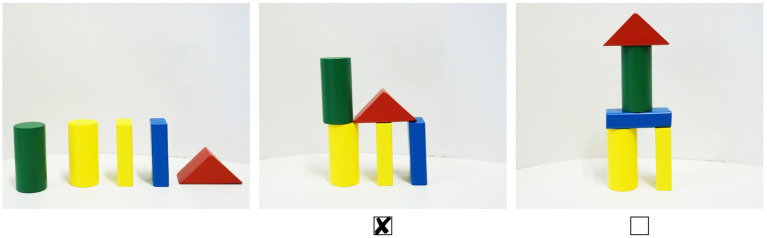
Multiple choice test: The correct spatial arrangement of the building blocks according to the rules had to be marked.

**Figure 4:**
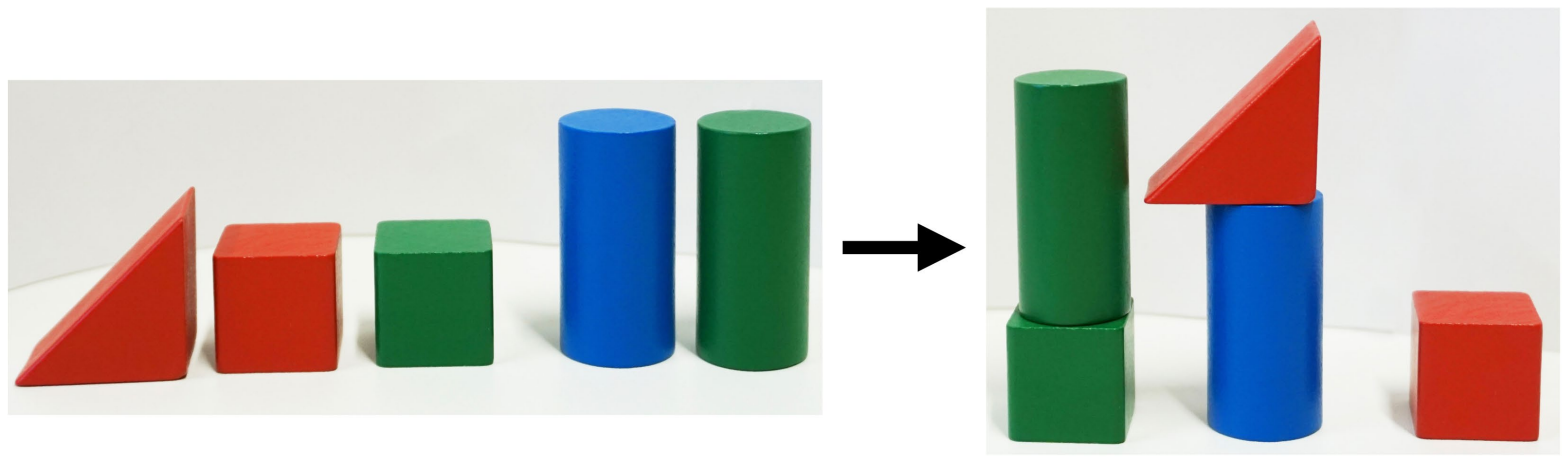
Construction test: The building blocks had to be arranged according to a particular rule or a combination of rules.

After the learning session, each participant performed a recognition test consisting of a multiple-choice questionnaire (MCQ) and a construction test (CT). Each test only contained one of the two rule sets, requiring participants to complete either the MCQ based on rule set A and the CT based on rule set B or vice versa. The MCQ and CT each consisted of eight tasks containing all rules of the respective set. Some tasks required a combination of rules to be applied, while others only involved particular rules. Each task of the MCQ consisted of three pictures: The first picture showed five building blocks, and the other two pictures either presented a wrong or a correct arrangement of the building blocks. The picture showing the correct arrangement according to the rules had to be marked (see Figure 3). In the CT, five different building blocks had to be arranged according to a particular rule or a combination of rules (see Figure 4). The order in which the two tests had to be completed was randomized over all participants.

On the second day of the study, a free recall task (FR) had to be performed. In the FR, participants were instructed to arrange a set of 38 building blocks according to as many rules as they could recall.

Participants received one point for each correctly solved task in the MCQ and the CT and for every correctly recalled and applied rule in the FR. Thus, they could score a maximum of eight points in both the MCQ and the CT and a maximum of ten points in the FR.

### 2.3. Statistical analysis

Statistical analysis was conducted with IBM SPSS Statistics (version 27). The Kolmogorov-Smirnoff-Test with Lilliefors correction revealed violations of the normal distribution concerning all variables and groups (all *D*s > 0.16, all *p*s < 0.05, see Supplementary Table S2 for details). Therefore, the Kruskal-Wallis test was used for the statistical analysis. The requirements of variance homogeneity and sphericity were fulfilled with *p* > 0.10. Consecutive Mann–Whitney *U*-tests were used for pairwise group comparisons.

## 3. Results

None of the groups differed significantly with respect to their grade of final secondary school examinations (*H(2) = 4.17, p = 0.124)*, and the rule sets A and B resulted in equivalent memory performance outcomes regarding the respective memory tasks (MCQ: *U* = 727.0, *z* = −1.04, *p* = 0.30; CT: *U* = 801.0, *z* = −0.31, *p* = 0.741; FR: *U* = 811.5, *z* = −0.23, *p* = 0.841). Consequently, differences between groups cannot be traced back to differences in rule sets.

**Figure 5:**
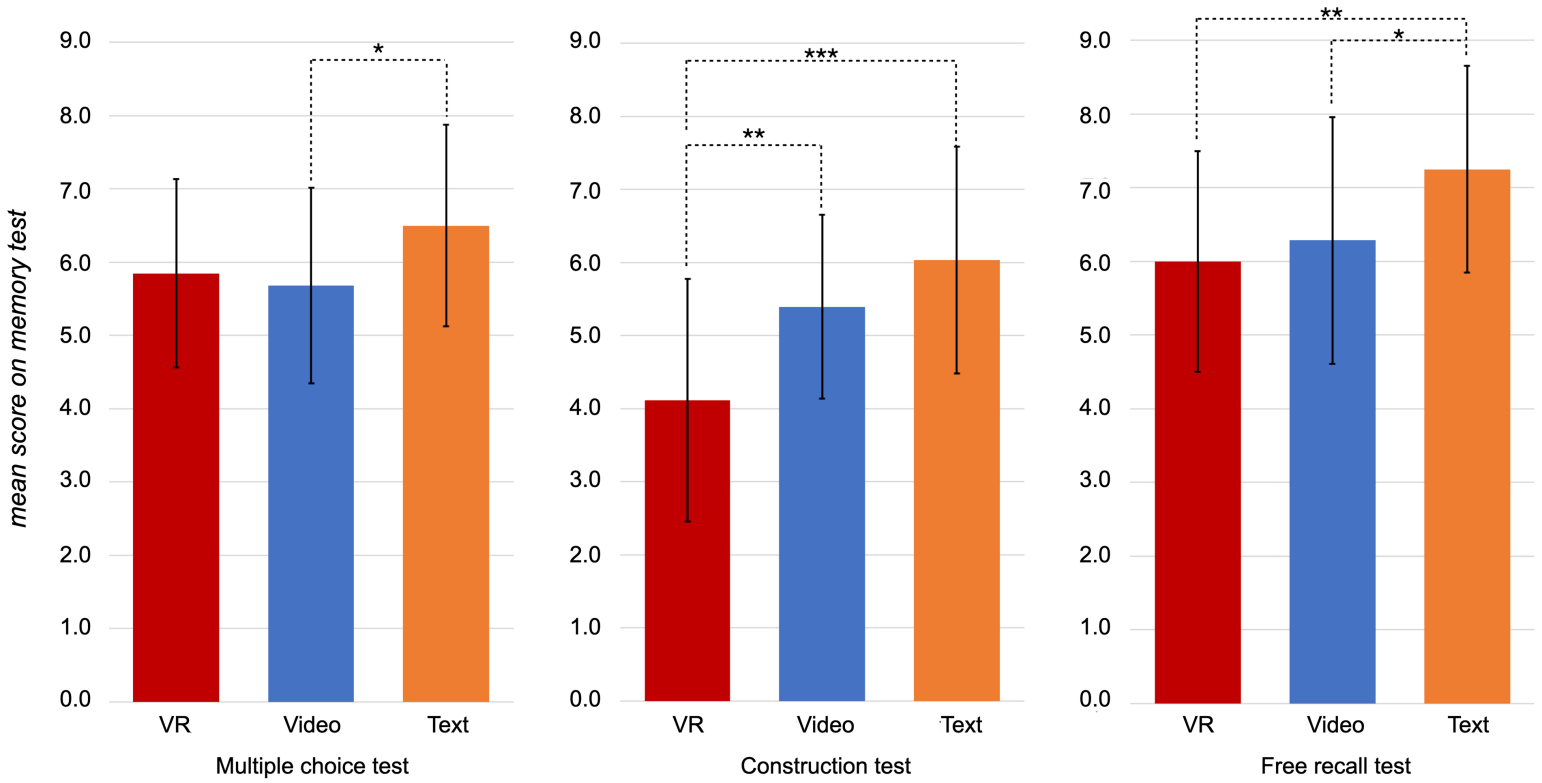
Mean scores of the achieved points in the respective memory task per group. The error bars depict the standard deviation from group mean. Significant differences between groups are marked, respectively, (* *p* < 0.05, ** *p* < 0.01, *** *p* < 0.001).

Regarding memory performance, the three groups performed significantly different on the memory tasks (MCQ: *H(2) = 6.06, p = 0.048;* CT: *H(2) = 17.01, p < 0.001*; FR: *H(2) = 9.52, p = 0.009*). In detail, the VR group and the video group performed equally well on the MCQ (*U* = 340.0, *z* = −0.43, *p* = 0.669, *r* = 0.059) and the FR (*U* = 341.5, *z* = −0.40, *p* = 0.689, *r* = 0.054). However, the video group outperformed the VR group in the CT (*U* = 200.5, *z* = −2.88, *p* = 0.004, *r* = 0.39; see Figure 5). Moreover, the text group performed better on both CT (*U* = 150.5, *z* = −3.74, *p* < 0.001, *r* = 0.51) and FR (*U* = 200.0, *z* = −2.90, *p* = 0.004, *r* = 0.40) compared to the VR group (see Figure 5). No significant differences were found between both groups concerning the MCQ (*U* = 259.0, *z* = −1.86, *p* = 0.063, *r* = 0.25). In line, text group and video group performed equally well on the CT (*U* = 279.5, *z* = −1.89, *p* = 0.059, *r* = 0.25) but the text group outperformed the video group in the MCQ (*U* = 255.0, *z* = −2.30, *p* = 0.021, *r* = 0.31) and the FR (*U* = 250.0, *z* = −2.37, *p* = 0.018, *r* = 0.32; see Figure 5).

## 4. Discussion

The present study aimed to further clarify under which conditions the use of VR can enhance mnemonic processing by comparing memory performance after learning information about the spatial arrangement of building blocks in VR, with a 2D video or a written text. Contrary to our expectations, the text condition provided the best overall results regarding memory performance, outperforming the VR condition in both the CT and the FR and the video condition in the MCQ and the FR. Additionally, the video condition outperformed the VR condition in the CT, whereas no superiority effect of the VR condition was observed at all.

In contrast to our results, several previous studies comparing text-, computer- and VR-based learning observed superiority effects for learning in virtual environments (Allcoat and von Mühlenen, 2018; Sattar et al., 2020; Zinchenko et al., 2020). However, this superiority often refers to factors such as motivation and engagement instead of actual task performance (e.g., Sattar et al., 2020). Although learning motivation is closely related to performance (Sankaran and Bui, 2001), several other factors influence learning and memory performance (e.g., Jackson, 2002; Cheng, 2011; Ulstad et al., 2016). Therefore, it is relevant to directly assess learning outcomes to evaluate educational applications (Radianti et al., 2020). Using virtual environments in education may thus have a positive influence on the learning process itself but does not necessarily lead to better performance outcomes when directly comparing different learning methods. Nevertheless, depending on individual motivational prerequisites, it is reasonable to apply VR in educational contexts to improve motivation and therefore learning performance, especially when learning engagement is low.

Interestingly, memories from VR experiences tend to become part of an autobiographical associative network (Schöne et al., 2019) and are therefore more effortlessly recalled than memories obtained from conventional 2D videos (Kisker et al., 2021c). These findings suggest that the memory for whole experiences benefits from VR. However, our data indicate that learning in VR is less beneficial for particular declarative information without relevant relation to the context it is presented in. In our study, listening to the rules in VR can improve memory for the experience itself, whereas it impedes focusing on declarative information about the rules. The surrounding nature of VR fosters presence, i.e., the feeling of physically being in the environment (Sanchez-Vives and Slater, 2005), which might increase the self-relevance of the situation (Kisker et al., 2021c). The consequently more salient stimuli automatically capture attention (Schöne et al., 2021b), leaving fewer resources for focusing on a specific content, which makes it harder to only concentrate on the rules regarding the spatial arrangement of the building blocks.

Moreover, VR creates realistic scenarios that elicit lifelike behavioral, psychophysiological, and affective reactions (Kisker et al., 2021a,d). The confrontation with the real-sized instructors in the VR condition generates a stronger perception of a social interaction leading to realistic reactions. Realistic social stimuli such as people or faces engage attentional resources while reducing the focus on other environmental aspects (Laidlaw et al., 2011). Divided attention in VR, resulting from the higher salience and social relevance of the stimuli, promotes the perception of multiple stimuli relevant to the overall virtual experience but hinders the learning of solely specific declarative information within that situation. Therefore, depending on the learning objective, VR can both enhance and impair mnemonic processing.

Overall, the participants in the text condition showed the best memory performance. Especially in the FR, they outperformed the participants in the other two conditions. Since the FR requires the real-life application of the learned information and assesses longer-term retention, it is the most relevant test for measuring learning outcomes in our study. The sample in our study consisted primarily of university students, and since lectures based on text slides are the standard method used in higher education (Kernbach et al., 2015; Roberts, 2019), students have to prepare for exams studying text-based material and are therefore used to this learning method. In contrast, the novelty of VR might be distracting and interfere with focusing on processing task-relevant information, an effect that might decrease as users become habituated to VR in educational contexts (Parong and Mayer, 2021). Future research should therefore investigate whether VR can be a more efficient tool for learning processes when it is used regularly and is established in the educational system.

Although the participants in the text condition had the same amount of time to learn the rules regarding the spatial arrangement of the building blocks as the other participants, they were able to actively contribute to their individual learning process. In contrast to the other participants, they could influence the pace at which they invested in learning specific information and could therefore read several rules repeatedly. Repetition-based learning enhances recall performance (Bromage and Mayer, 1986), and the presented content is better understood when participants have control over the presentation pace (Mayer and Moreno, 2003; Wahl et al., 2022) which might explain the superior memory performance in the text group. Additionally, in comparison to the learning session in our study, studies that found a memory superiority effect of VR used tasks in which the participants were actively engaged in the learning process in the VR condition as well (Allcoat and von Mühlenen, 2018; Krokos et al., 2019; Zinchenko et al., 2020). For example, participants could interact with a 3D model in VR and resize, highlight or rotate individual parts of the 3D model during learning (Allcoat and von Mühlenen, 2018). In contrast, participants in our study received the information about the rules by passively watching the instructions *via* a 2D or a 3D/360° video, without the option to influence the pace or to actively contribute to the learning process. Individual active learning is a more effective learning method than passive learning (Riley and Ward, 2017), and active manipulation in VR yields better learning outcomes than passive viewing (Jang et al., 2017). Participants actively controlling the learning process felt more immersed in a virtual scene than in a desktop scene and were more focused on the task (Krokos et al., 2019). Hence, when actively focusing on a given task, VR does not always hinder concentration on relevant information but enhances it through stronger immersion. Which environmental factors in virtual compared to conventional educational contexts promote or impair focusing on the learning task is the subject of future research. However, active involvement promotes learning outcomes and should be pursued when applying new technologies in educational contexts.

The effectiveness of using virtual environments also depends on the domain in which it is applied. For understanding and learning complex and detailed information such as plant cell or heart structures, the improved visualization through three-dimensional models was observed to be advantageous for memory performance measured with knowledge tests (Allcoat and von Mühlenen, 2018; Zinchenko et al., 2020). In contrast, the benefit of this improved visualization is attenuated for more simplistic stimuli such as the building blocks used in our study. The three-dimensional visualization of the building blocks does not contribute to comprehending the declarative information regarding the rules and therefore does not improve information processing in this task. Additionally, taking the learner’s perspective into consideration, the application of VR is particularly recommended for learning areas in which information can be learned by shifting into another person’s perspective, by experiencing social situations, by learning in environments that would not be accessible in reality or by actively involving the learner (Wahl et al., 2022). However, for declarative knowledge acquisition that does not require understanding three-dimensional structures or relations, conventional learning methods are sufficient, whereas a superiority effect of VR emerges for understanding and learning more detailed and complex information through realistic visualization that is not feasible in reality and meets the learner’s needs.

Although our data suggest differences in memory performance between the three conditions, the sample size has to be taken into consideration. Due to the relatively small number of participants in each condition, smaller effects might not have been detected. Additionally, only two answer choices were given in the MCQ, which leads to a high guessing probability in all three conditions. Therefore, even more differences between the conditions are conceivable than those observed in our study.

In existing work on learning in VR, educational research findings have been synthesized to a theoretical framework that describes the influence of different factors on knowledge acquisition and transfer (Makransky and Petersen, 2021). Correspondingly, a theoretical framework has to be developed for general mnemonic processing in VR going beyond the educational context. Since first evidence suggests that previous findings regarding memory processes cannot be completely transferred to VR experiences (Kisker et al., 2021c) and that memory formation in VR differs from the conventional laboratory (Schöne et al., 2019), a comprehensive overview over the data is necessary. Therefore, our study is a first step in generating data for developing a theoretical background applicable on general mnemonic processing.

Taken together, although new technologies are promising tools to improve mnemonic processes, several factors should be considered when applying virtual environments. The effect of VR on memory performance varies depending on several factors, such as the learning objective, the learning task, individual learning prerequisites, and the domain it is applied on. The precise circumstances under which VR leads to efficient mnemonic processing and should thus be applied in educational contexts remain to be further specified. Learning outcomes in domains that benefit from enhanced three-dimensional visualization can be improved by using active learning tasks in VR that facilitate focusing on relevant information. However, our study suggests that the affordances of VR cannot always be leveraged for accomplishing the final objective of education, the application of learned knowledge in different contexts (Bossard et al., 2008). Conventional learning methods are sufficient for learning and applying basic declarative information, and therefore the effort to develop an equivalent learning environment in VR is not always profitable. Nevertheless, it should be noted that the results of our study refer to this specific task and their generalizability to other learning tasks remains to be verified. In particular, as other studies have already provided evidence for a memory superiority effect of VR content over a conventional screen presentation (Schöne et al., 2019; Kisker et al., 2021b).

## Data availability statement

The datasets presented in this study can be found in online repositories. The names of the repository/repositories and accession number (s) can be found at: OSF: https://osf.io/pwtsa/?view_only=d136e0e2b56e461e9a287adfd52d0da2

## Ethics statement

The studies involving human participants were reviewed and approved by the local ethic committee of Osnabrück University, Germany. The patients/participants provided their written informed consent to participate in this study.

## Author contributions

BS, KAP, TS, VLT, and AW created the stimulus material and acquired the data. Data analysis was performed by JK. MJ drafted the manuscript under the supervision of BS. TG provided critical revisions. All authors contributed to the article and approved the submitted version.

## Funding

This research was funded by the MWK Niedersachsen and the VolkswagenStiftung (grant number 11-76251-14-1/21). We acknowledge support by Deutsche Forschungsgemeinschaft (DFG) and Open Access Publishing Fund of Osnabrück University.

## Conflict of interest

The authors declare that the research was conducted in the absence of any commercial or financial relationships that could be construed as a potential conflict of interest.

## Publisher’s note

All claims expressed in this article are solely those of the authors and do not necessarily represent those of their affiliated organizations, or those of the publisher, the editors and the reviewers. Any product that may be evaluated in this article, or claim that may be made by its manufacturer, is not guaranteed or endorsed by the publisher.

## Footnotes

^1^ https://www.insta360.com/download/insta360-pro%3finspm%3d77c1c2.89f76e.0.0
