# Supplementary material for: New is not always better: Virtual reality does not necessarily enhance mnemonic processing
Source: Front Psychol. 2023 Feb 15;14:1089725. doi: 10.3389/fpsyg.2023.1089725 (PMC9975255; doi:10.3389/fpsyg.2023.1089725)
Supplement: Supplementary file 1 [file Data_Sheet_1.pdf]

Supplementary Material

Table S1

Rule sets for the arrangement of the building blocks.

set	rule
set A	A1. Cuboids have to stand on their smallest side.
	A2. On cuboids there must always lie a roof.
	A3. Green flat cuboids must always lay on their biggest side.
	A4. Red cylinders must always stand on a flat cuboid.
	A5. Two blue objects must touch each other.
set B	B1. Flat cuboids have to stand on their smallest side.
	B2. Flat cuboids must always stand on a cube.
	B3. Yellow tetrahedrons must always lay on their smallest side.
	B4. Blue cylinders must always touch a tetrahedron.
	B5. Two green objects must touch each other.

Table S2

Kolmogorov-Smirnov test with Lilliefors correction for normal distribution: test statistics.

		descriptive statistics			Kolmogorov-Smirnov test with Lilliefors correction for normal distribution		
variable	group	*Mean*	*Md*	*SD*	*D*	*df*	*p*
grade of final secondary-school examinations	VR	1.48	1.45	0.3	0.161	26	.066
	Video	1.70	1.50	0.62	0.250	28	<.001
	Text	1.41	1.40	0.36	0.262	28	<.001
Multiple choice	VR	5.85	6.00	1.29	0.163	26	0.074
	Video	5.68	6.00	1.33	0.238	28	<.001
	Text	6.50	7.00	1.37	0.213	28	.002
Construction test	VR	4.12	4.00	1.66	0.211	26	.004
	Video	5.39	5.50	1.26	0.185	28	.015
	Text	6.04	6.00	1.55	0.169	28	.039
Free Recall	VR	6.00	6.00	1.50	0.192	26	.014
	Video	6.29	6.00	1.67	0.211	28	.003
	Text	7.25	7.00	1.40	0.179	28	.021
